# Future Training and Competency Needs in Hospital‐at‐Home Care: A Mixed‐Methods Study From the Perspective of Healthcare Administrators and Managers

**DOI:** 10.1155/jonm/3461359

**Published:** 2026-04-22

**Authors:** Isakov Terhi-Maija, Härkönen Henna, Jansson Miia

**Affiliations:** ^1^ Research Unit of Health Sciences and Technology, University of Oulu, Oulu, Finland, oulu.fi; ^2^ Medical Research Center Oulu, Oulu, Finland, oulu.fi; ^3^ STEM College, Royal Melbourne Institute of Technology (RMIT University), Melbourne, Australia

**Keywords:** competency, health care professionals, home-based healthcare, hospital-at-home, mixed-methods research

## Abstract

**Background:**

The growing complexity of hospital‐at‐home care highlights the pressing need for continuous professional development to enhance specialized skills.

**Aim:**

This study aimed to describe the future training and competency needs in hospital‐at‐home care from the perspective of healthcare administrators and managers.

**Design:**

A mixed‐method convergent design was used to integrate quantitative and qualitative data on similar topics simultaneously, providing a more comprehensive understanding of system‐level competency requirements than either method alone.

**Methods:**

The data were collected through a nationwide survey (*n* = 25) and interviews (*n* = 46), covering all wellbeing services counties in Finland in 2023. Descriptive statistics were used for quantitative data and inductive content analysis for qualitative data.

**Results:**

Quantitative results indicate needs for additional training in RAI assessment (68%), mental health care (60%), substance abuse expertise (48%), and managing disruptive behaviors (44%). Qualitative findings highlight broader future competence needs, including digital health; cultural and linguistic competence; interprofessional collaboration; managerial competence; home‐environment quality and safety; clinical competence; and autonomy in evidence‐based practice. Overall, the mixed‐method findings differ yet complement one another: quantitative data identify concrete skill gaps, while qualitative insights emphasize wider, system‐level competencies to meet the evolving demands of home‐based care.

**Conclusion:**

Taken together, these complementary findings indicate that advancing the workforce will require a dual approach: addressing concrete skill deficits while simultaneously developing the broader competencies needed to meet the evolving demands of home‐based care.

**Implications for Nursing Management:**

This study informs the creation of structured competency frameworks, enabling nursing leaders to meet the evolving demands of home‐based care.

## 1. Introduction

Hospital‐at‐home (HaH) is a short‐term, targeted service that delivers hospital‐level care in a patient’s home, ensuring the same quality of treatment as in a traditional hospital setting. As an evolving model, HaH has the potential to transform healthcare delivery. However, the increasing complexity of healthcare services has expanded the roles and responsibilities of professionals [1, 2]. In response, healthcare organizations are prioritizing structured competence training and workforce development to uphold high standards of patient care [3].

In nursing practice, competence encompasses the comprehensive ability of health professionals to perform their roles effectively and professionally, integrating essential knowledge, skills, attitudes, values, and performance [4]. It includes both theoretical understanding and practical application [5]. Competency, on the other hand, refers to specific, measurable skills and knowledge required for professional duties. International frameworks define core competencies for registered nurses [6, 7], with specialized competency standards for areas such as critical care [8].

The professional workforce delivering HaH care varies internationally. However, nurses represent the largest professional group in HaH care [9] and often practice independently when delivering care in patients’ homes. A recent study by Rusli et al. [10] identified 10 essential competencies for home‐based nursing care: care assessments, nursing procedures, health condition management, critical thinking and problem‐solving, interpersonal communication, interdisciplinary collaboration, leadership and resource management, professional development, technological literacy, and quality and safety. Although this review provides a thorough overview of nursing competencies, it lacks specificity regarding the requirements of other healthcare professionals involved in HaH care, including physicians, licensed practical nurses, social workers, and allied health therapists.

The implementation and delivery of HaH services vary considerably across countries, underscoring the need for context‐specific approaches to expanding workforce competencies. While some settings have established competency frameworks [11], others are in the process of developing them [12], and in many regions, such frameworks are still absent.

In Finland, HaH services emerged in the early 1990s. Today, nurse‐led HaH programs cover approximately 92% of the population [13]. However, their continued development is hindered by the absence of standardized definitions and by substantial variation in service models across wellbeing services counties. These inconsistencies pose significant challenges for determining and planning workforce competencies, particularly those required for clinical assessment and decision‐making in home‐based care.

Achieving truly integrated, multidisciplinary care requires a deeper understanding of the evolving training and competency needs across all professional groups within home‐based healthcare. To address this gap, we conducted a mixed‐methods study exploring the key future training and competency needs in HaH care from the perspective of healthcare administrators and managers. Their perspectives are critical, as these leaders are directly responsible for strategic workforce planning, resource allocation, and the implementation of new models of care. Consequently, their insights provide an essential foundation for identifying system‐level competency needs and guiding the development of effective training strategies.

Quantitative data allowed us to identify the level of current competencies and perceived competency needs across wellbeing services counties, whereas qualitative data provided the contextual depth needed to understand how administrators and managers interpret these needs within their operational, organizational, and regional realities. Integrating these complementary strands provided a more comprehensive understanding of system‐level competency requirements than either method alone. The study was guided by the following research questions: (1) *What are the key training needs in HaH care* and (2) *What are the future competency needs in HaH care?*

## 2. Methods

### 2.1. Study Design

A mixed‐methods convergent design was used to integrate quantitative and qualitative data on similar topics simultaneously [14]. The study was reported according to the Checklist of Mixed Methods Elements in a Submission to Advance the Methodology of Mixed Methods Research [15].

### 2.2. Participant Selection and Recruitment

The study was conducted across all Finnish wellbeing services counties and encompassed 22 public and 3 private healthcare providers between August and October 2023, thereby constituting a complete (total) sample. A nonprobability sampling strategy was employed, whereby participants most relevant to the study’s aims were identified through the official websites of the wellbeing services counties (purposive sampling). Recruitment was carried out by the doctoral researcher (M.Sc. H.H.), who had no prior relationship with the participants.

Administrators and managers involved in HaH activities were contacted via email or telephone and invited to participate in a nationwide cross‐sectional survey, followed by supplementary interviews. To be eligible, participants had to be directly involved in the management and delivery of HaH services, with knowledge of or access to their organization’s resource management. If deemed ineligible, participants were encouraged to recommend others for further contact.

This study was conducted in Finland, where HaH services are typically provided in Finnish or Swedish, with residents of Nordic, EU, EEA countries, and Switzerland entitled to receive care in their native language. If necessary, interpretation and translation services are arranged for patients arriving from other countries. The most common indications for HaH care include infectious diseases, as well as palliative and end‐of‐life care [4].

In Finland, the Resident Assessment Instrument (RAI) is commonly used to assess clients in home and long‐term care, especially older adults, providing consistent and comprehensive data on their health status and service needs [16]. While Finland’s healthcare system is highly digitized, digital maturity varies between primary and specialty care [17]. Notably, ambulatory and home care services show a higher level of digital maturity compared to HaH care [18].

### 2.3. Data Collection

**FIGURE 1:**
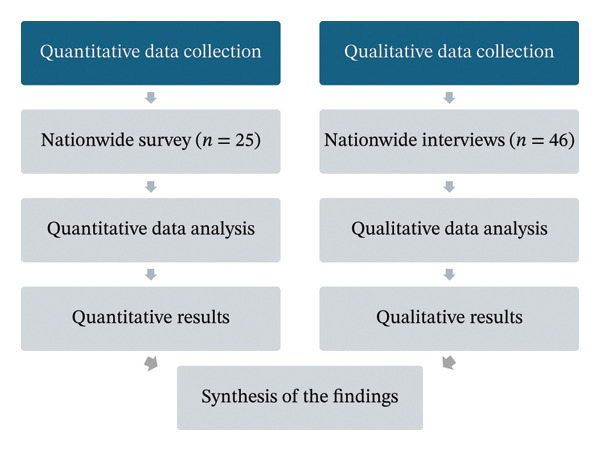
A procedural diagram of the data collection and analysis procedures.

The data were collected through nationwide survey and interviews (Figure 1). Each wellbeing services county provided one centralized response to the survey, whereas one to two participants per county were allowed to take part in the interview.

#### 2.3.1. Cross‐Sectional, Nationwide Survey

Participants received an email containing a personalized link to a previously developed, structured, self‐administered multiple‐choice questionnaire (available from the original developers upon request) [19]. Training needs were evaluated using four background questions and 23 predefined competence areas, rated on a three‐point scale (good, moderate, or requiring further training). Respondents were asked to rate the competency of their current employees in these areas with the question: “*Please rate the competency of your current employees in the following areas*”. Before submitting, participants had the option to save and edit their responses, and they could also select multiple answer choices. The interview framework was pretested in one group interview and one individual interview. As the framework proved effective, no modifications were necessary. The data collected during pretesting were included in the final analysis.

#### 2.3.2. Semistructured, Nationwide Interviews

Nationwide interviews were conducted with 46 participants between August and October 2023. All interviews were conducted remotely in Finnish via Microsoft Teams (Microsoft Corporation, Redmond, WA). In total, 25 semistructured interviews were completed, including 21 pair interviews and 4 individual interviews. All interviews were conducted by two researchers (M.Sc. H.H. and PhD M.J.) with methodological expertise.

Future competence needs were examined as part of a broader interview study exploring the current state of HaH services in Finland [13]. The interview guide included open‐ended questions addressing the delivery, organization, coordination, resourcing, and overall impact of HaH services. It also incorporated questions related to the professional workforce and evolving competency requirements, such as: *“What kinds of professional groups and job descriptions does HaH care require now and in the future?”* Probing questions (e.g., *“Could you elaborate on that?”*) were used to elicit deeper, more nuanced insights.

All sessions were audio‐recorded and transcribed verbatim by the first author (PhD T‐M.I.). All interviews were conducted by one researcher (M.H.) and were audio‐recorded. The interviews lasted an average of 34 min, with durations ranging from 21 to 42 min.

### 2.4. Data Analysis

The quantitative data were stored in password‐protected Microsoft Excel spreadsheets. Descriptive statistics, including frequency distributions and percentages, were used to summarize the distribution of training needs and responses to the background questions.

The software package NVivo 12 Plus for Windows was used for qualitative data (coding, grouping, categorization, and abstraction). The qualitative dataset comprised 781 pages with 1.08 line spacing and 12 pt Calibri font. Before analysis began, sentences were defined as the unit of analysis, and inductive content analysis was conducted [20]. The data were read multiple times for a comprehensive understanding, after which 198 codes were identified. These codes were then organized into subcategories, general categories, and main categories, each named using content‐specific terms.

Synthesis of the findings was conducted narratively after completing the quantitative and qualitative analyses, comparing results from both strands to generate a more comprehensive understanding of the research problem.

### 2.5. Ethical Considerations

This research was conducted in accordance with the Declaration of Helsinki (1964). As per Finnish legislation (Medical Research Act no. 488/1999), research ethics committee approval was not required, as the study did not involve minors, direct or indirect physical or physiological harm to participants, or clinical trials. Approval was granted by the relevant academic center in 2023. Written informed consent was obtained from all participants prior to their involvement, and permission to record interviews was secured from each individual. Data were handled in compliance with the European Union’s General Data Protection Regulation (2018).

### 2.6. Rigor and Reflexivity

In the quantitative section, the reliability of the questionnaire was ensured by adapting it from a previously validated instrument [19], which has been used in Finland since 2014 to monitor the implementation of the Elderly Services Act, the Quality Recommendation, and key national development projects. Since the competencies in the questionnaire were based on the survey of elderly care services, a qualitative interview was also conducted to gain deeper insights into the specific competence requirements for HaH care.

The trustworthiness of the qualitative section was evaluated using established qualitative research standards, including dependability, credibility, confirmability, transferability [21], and authenticity [22]. To ensure dependability, a rigorous data collection and analysis process was implemented. Confirmability was enhanced through triangulation, with data collected by two researchers (M.Sc. H.H. and PhD M.J.) and analyzed by one (PhD T‐M.I.), with validation conducted by two researchers (M.Sc. H.H. and PhD M.J.). Transferability was supported by providing a detailed description of the study characteristics and analysis. Authenticity was maintained by using the participants’ original expressions. Data saturation was monitored by two researchers and was deemed achieved when further interviews no longer revealed new insights into the phenomenon being studied [20].

## 3. Results

### 3.1. Demographic Characteristics

The interview participants (*n* = 46) were primarily from the public sector (89.1%) and predominantly (89.1%) female (Table 1). In terms of roles, 17.4% were in senior management, 47.8% in middle management, 30.4% in frontline management, and 4.4% in project/expert roles (Table 2). Contrary to the public sector, the private sector faced a shortage of specialized nursing expertise in hospice and palliative care. All recruited participants completed the study without any withdrawals.

**TABLE 1:** Demographics of the interview’s participants.

Demographics of the interviews’ participants (*n* = 46)	No. (%)
Organizational sector	
Public	41 (89.1)
Private	5 (10.9)
Gender	
Female	41 (89.1)
Male	5 (10.9)
Position	
Senior management	8 (17.4)
Middle management	22 (47.8)
Frontline management	14 (30.4)
Project/expert work	2 (4.4)

**TABLE 2:** Provision of hospital‐at‐home (HaH) care specialization in wellbeing services counties (*n* = 25).

	**No (%)**

Organizer of HaH care in the wellbeing services county	
Public	19 (76.0)
Private	0 (0.0)
Both	6 (24.0)
Available hospice/palliative care volunteers care^∗^	
Yes	16 (69.6)
No	7 (30.4)
Specialization (physicians)	
No specialization	2 (8.0)
General medicine	16 (64.0)
Geriatrics	15 (60.0)
Internal medicine	5 (20.0)
Palliative medicine	20 (80.0)
Pain medicine	0 (0.0)
Other (e.g., anesthesiology, acute care, psychiatry)	8 (32.0)
Specialization (nurses)	
No specialization	1 (4.2)
Diabetes	9 (37.5)
Endoprosthesis	1 (4.2)
Wound care	11 (45.8)
Hospice and palliative care	21 (87.5)
Other	10 (41.7)

^∗^missing data.

### 3.2. Cross‐Sectional, Nationwide Survey of Training Needs

Quantitative results indicate needs for additional training in RAI assessment (68%), mental health care (60%), substance abuse expertise (48%), and managing disruptive behaviors (44%). Competencies like palliative care (72%) and pharmacological treatment (92%) were already well‐covered, with fewer providers reporting a need for further training. The majority of providers (96%) expressed confidence in professionals’ competence in engaging with clients and their relatives (Table 3). These findings align with the competencies identified in the interviews, including clinical and digital health competencies, cultural and religious sensitivity, and ensuring quality and patient safety within the home environment.

**TABLE 3:** Healthcare providers’ perceived training needs across various competency areas in both public and private healthcare sectors within Finnish wellbeing service counties (the participants were permitted to select multiple response options).

Competency areas	Good, *n* (%)	Moderate, *n* (%)	Requiring further training, *n* (%)
RAI assessment^∗^	0 (0.0)	5 (20.0)	17 (68.0)
Mental health	3 (12.0)	10 (40.0)	15 (60.0)
Substance abuse expertise	2 (8.0)	12 (48.0)	12 (48.0)
Management of patients exhibiting disruptive behavior	2 (8.0)	11 (44.0)	11 (44.0)
Use of information systems	13 (52.0)	6 (24.0)	9 (36.0)
Supporting customers in the use of technology	2 (8.0)	13 (52.0)	9 (36.0)
Oral health care	5 (20.0)	13 (52.0)	9 (36.0)
Multidimensional assessment of functional capacity	5 (20.0)	11 (44.0)	8 (32.0)
Using technology to support nursing	6 (24.0)	14 (56.0)	8 (32.0)
Identification of existential and spiritual needs	9 (36.0)	11 (44.0)	7 (28.0)
Supporting the autonomy of people with memory loss	6 (24.0)	12 (48.0)	7 (24.0)
Rehabilitation approach	6 (24.0)	14 (56.0)	7 (28.0)
Palliative and hospice care	18 (72.0)	3 (12.0)	6 (24.0)
Real‐time electronic documentation	11 (48.0)	11 (44.0)	5 (20.0)
Nonpharmacological treatment	10 (40.0)	10 (40.0)	5 (20.0)
Identification of psychosocial symptoms^∗^	10 (40.0)	12 (48.0)	4 (16.0)
Meeting loved ones at the approach of death and in bereavement	18 (72.0)	3 (12.0)	4 (16.0)
Nutrition	13 (52.0)	10 (40.0)	3 (12.0)
Fall prevention	9 (36.0)	15 (60.0)	2 (8.0)
Pharmacological treatment	23 (92.0)	1 (4.0)	2 (8.0)
Other symptoms’ treatment (e.g., shortness of breath, constipation)	22 (88.0)	1 (12.0)	2 (8.0)
Pain treatment	20 (80.0)	3 (12.0)	2 (8.0)
Meeting clients and relatives	24 (96.0)	0 (0.0)	1 (4.0)

^∗^missing data.

### 3.3. Semistructured, Nationwide Interviews of Competency Needs

The future competence needs were organized into 7 main categories, 18 generic categories, and 51 subcategories. These main categories included digital health; cultural and linguistic competence; interprofessional collaboration; managerial competence; home‐environment quality and safety; clinical competence; and autonomy in evidence‐based practice (Table 4).

**TABLE 4:** Categorization of the future competency needs in hospital‐at‐home (HaH) care.

Subcategory	Generic category	Main category
Knowledge of using technology in clinical practice	Utilization of Technology in Clinical Practice	Digital Health Competence
Skills to use technology in clinical practice		
Attitude toward using technology in clinical practice		
Providing customer support in the context of technology use		
Skills in using information systems	Utilization of Information Systems in Clinical Practice	
Negative attitudes toward the use of information systems		

Finnish language proficiency for immigrant nurses	Linguistic Skills	Cultural and Linguistic Competence
Language barriers between Finnish professionals and foreign patients		
Interpreting expressions	Cultural and Religious Sensitivity	
Addressing religious needs		
Understanding cultural norms		

Two‐way communication between service providers	Interdisciplinary Collaboration	Interprofessional Collaboration Competence
Work distribution between service providers		
Cooperation between service providers		
Trust between physicians and nurses in remote care settings		
Division of duties between registered and practical care nurses	Interpersonal Collaboration	
Pedagogical competence	Knowledge Sharing	
Remote support		

Work rotation management	Talent Management	Managerial Competence
Need‐based care skills development		
Competence management		
Leading remote teams	Human Resources Management and Leadership	
Adjusting staffing levels and workload		
Strategic management	Business Management	
Financial management		

Hazards identification	Occupational Safety During Home Visits	Home Environment Quality and Safety Competence
Risk assessment		
Occupational safety management		
Coping with unhygienic conditions	Ensuring Quality and Patient Safety within Home Environment	
Ensuring patient safety		

Pharmacological and nonpharmacological pain management	Clinical Treatment Skills	Clinical Competence
Rehabilitation		
Intravenous therapy		
Wound care		
Expertise from allied health professionals	Advanced Clinical Expertise	
Expertise from gerontology		
Expertise from palliative and hospice care		
Care for diverse patient populations	Comprehensive Clinical Knowledge	
In‐depth understanding of health and social care systems		
Skills for providing psycho‐social support		
Use of a broad spectrum of professional expertise		

Preference autonomous work	Independent Work Ability	Autonomy in Evidence‐Based Practice
Formal and informal qualifications		
Experience required for independent practice		
Willingness to work independently in diverse environment	Professional Role and Self‐Development Attitude	
Willingness to work independently in complex environments		
Ability to learn skills beyond one’s primary profession		
Care assessment skills	Ability to Assess and Apply Evidence‐Based Practices	
Triage skills		
Cognitive and functional assessment skills		
Evidence‐based decision‐making skills		

#### 3.3.1. Digital Health Competence

Digital health competence was divided into two main categories: Utilization of technology and Utilization of information systems in clinical practice.

Utilization of technology in clinical practice was divided into four subcategories: knowledge of, skills to, and attitudes toward using technology in clinical practice, as well as providing customer support in the context of technology use. There is a growing need to incorporate more advanced technologies (e.g., smart thermostats, home security systems) into clinical practice. However, a significant gap in knowledge and skills exists for using these technologies effectively. This need was highlighted in one of the interviews:

*“However, we also face the challenge of understanding how to effectively use the existing technology, especially as more patients will have access to advanced smart technologies to support them in living at home.”* (Interview 1)

Attitudes toward the use of technology were somewhat negative, primarily due to the technical maintenance required to ensure the proper functioning of devices, such as vehicles. However, the need for technical support for patients has grown, as highlighted in one of the interviews:

“*People increasingly require more technical support these days, particularly when patients encounter issues with devices like pain pumps at home and need guidance on how to use them*.” (Interview 5)

Utilization of information systems in clinical practice was divided into two subcategories: skills in using information systems and negative attitudes (e.g., reluctance) toward their use. While training has improved skills in using information systems, there is still a need for further training, as highlighted by one participant:

“*It is worth noting that some individuals, including myself, lack the necessary IT skills despite the training provided*.” (Interview 14)

#### 3.3.2. Cultural and Linguistic Competence

Cultural and linguistic competence was divided into two generic categories: *Linguistical skills* and *Cultural and religious sensitivity*.

Linguistic skills were categorized into two subcategories: Finnish language proficiency for immigrant nurses and language barriers between Finnish healthcare professionals and foreign patients. Although interpretation services are available and many nurses are fluent in English, a significant number of patients speak languages other than English, posing communication challenges. To effectively meet the needs of a diverse patient population, a broader range of verbal and nonverbal communication skills is necessary:

“*Our clients come from diverse linguistic and cultural backgrounds, but what matters most is establishing a strong, meaningful connection with them*.” (Interview 6)

Cultural and religious sensitivity was divided into three subcategories: interpreting expressions, addressing religious needs, and understanding cultural norms. There is a need to interpret expressions of pain and discomfort in a way that is sensitive to the cultural nuances of each individual. Additionally, healthcare professionals must be mindful of the diverse religious needs, particularly when caring for terminally ill patients. Adapting to the cultural norms observed in the patient’s home also requires a high level of cultural sensitivity. This was illustrated in the following excerpt from an interview:

*“I must first obtain permission to carry out my work within the patient′s home. As a result, adapting to the prevailing cultural norms becomes a key aspect of my role*.” (Interview 11)

#### 3.3.3. Interprofessional Collaboration Competence

Interprofessional collaboration competence was divided into three generic categories: *Interdisciplinary collaboration*, *Interpersonal collaboration*, and *Knowledge sharing*.

Interdisciplinary collaboration was divided into four subcategories: improving two‐way communication, enhancing work distribution, fostering cooperation between service providers, and building trust between physicians and nurses in remote care settings:

“*It is based on respect and trust of other professional group, because nurses make decisions during visits and report changes in health status to the physician, who is not present*.” (Interview 5)

Interpersonal collaboration involved the division of duties between registered nurses and practical care nurses throughout the HaH pathway, including both HaH and residential services. The aim was for practical nurses to assist with home visits, providing basic care and improving the efficiency of these visits. Despite nurses in HaH care and those in residential services having comparable qualifications, they are not granted the same level of authority to practice—such as the ability to administer intravenous (IV) therapy. As one participant explained:

“*In cooperation with the nursing homes, the goal would, of course, be that the registered nurses in the nursing homes could participate in this medication treatment when the registered nurse is on duty, to make better use of the resource*.” (Interview 10)

Knowledge sharing was divided into two subcategories: pedagogical competence and remote support. There is an increasing demand for in‐hospital expertise in training other healthcare providers, particularly in specialized areas like tracheostomy management. Additionally, the demand for remote support in the use of medical devices is increasing, as highlighted in one interview:

“*I′ve noticed that we′ve become a low-threshold unit, where the HaH care team is now the first point of contact for provincial home care units. If they need training—whether it′s on pump usage, antibiotics, or other equipment—they reach out to us*.” (Interview 21)

#### 3.3.4. Managerial Competence

Managerial competence was divided into three generic categories: *Talent management*, *Human resources management and leadership*, and *Business management.*

Talent management was divided into three subcategories: work rotation, need‐based care skills development, and competence management (e.g., workforce development). There is a need to develop care skills based on patient needs by defining client profiles in advance and setting measurable competency requirements specific to HaH care. This is illustrated in the following interview:

“*We need to develop the skills to strengthen the internal capacity of HaH services. This includes client profiling, more precise definition of competency requirements, workforce development, and harmonizing work processes where appropriate, along with measuring the effectiveness of these activities*.” (Interview 15)

Human resources management and leadership were divided into two subcategories: leading remote teams and adjusting staffing levels and workload. Leading mobile workforces requires specialized skills, such as managing work across multiple locations and navigating different cultural contexts. Adjusting staffing levels and workload involves understanding travel times, task duration, and organizing work to minimize overlap while ensuring quality care with the appropriate competency, as highlighted in one interview:

“*We need the skills to ensure that routes and visits are logically planned, activities and staffing are well coordinated, employees feel competent, and work is organized across a wide area while maintaining high-quality care*.” (Interview 12)

Business management was divided into two subcategories: strategic management and financial management. Knowledge of strategic management is crucial in ensuring the sustainability of services. The need for knowledge of financial management is twofold: first, there is a requirement for strategic decision‐making to reduce costs and enhance profitability; second, there is a demand for practical guidance, including an understanding of pricing strategies:

“*Yes, financial management knowledge is crucial for handling these bills, but it′s important to remember that HaH care isn′t always less expensive. However, it is more cost-effective for the patient*.” (Interview 19)

#### 3.3.5. Home Environment Quality and Safety Competence

Home environment quality and safety competence was divided into two generic categories: *Occupational safety during home visits* and *Ensuring quality and patient safety within home environment.*

Occupational safety during home visits was divided into three subcategories: hazard identification, risk assessment, and occupational safety management. Hazards included ergonomic risks, work‐related violence, aggression, hostile animals, and unsafe or unsanitary conditions. Risk assessments should be conducted in advance to minimize healthcare professionals’ exposure to workplace injury and illness, with the possibility of adjusting care plans if needed. Occupational safety management included knowing safety protocols, adherence to guidelines, and recognizing when to involve a partner or security service for extra safety during home visits:

“*In cases of substance abuse, intravenous drug use, or other uncertain situations involving these substances, the safety of the employee can be at risk. Therefore, it is crucial to ensure occupational safety during home visits by being familiar with the proper protocols for responding to such situations*” (Interview 10)

Ensuring quality and patient safety within home environment was divided into two subcategories: managing unhygienic conditions and ensuring patient safety. Managing unhygienic conditions required knowledge and resourcefulness to maintain hygienic and sterile care environments, particularly for procedures like IV administration. Ensuring patient safety involved identifying potential risks, such as unsafe living conditions or fire hazards linked to oxygen therapy, and effectively reporting these risks to ensure patient well‐being:

*“When entering a new home with oxygen cylinders and identifying a fire safety risk or similar hazard, nurses must promptly report these concerns to the relevant authorities to ensure the safety of the patient and the environment*.” (Interview 1)

#### 3.3.6. Clinical Competence

Clinical competence was divided into three generic categories: *Clinical treatment skills*, *Advanced clinical expertise*, and *Comprehensive clinical knowledge.*

Clinical treatment skills were divided into four subcategories: pharmacological and nonpharmacological pain management, rehabilitation, IV therapy skills, and wound care. HaH care involves managing a wide range of medical conditions that would typically require hospitalization. Possessing strong clinical treatment skills is essential to ensure healthcare professionals can deliver comprehensive care in the home setting. However, due to the limited number of accredited wound care nurses, there is an increasing need to explore technical solutions to address this gap in the future, as highlighted in one interview:

“*We have only a limited number of accredited wound care nurses (---), so in the future, we may need to explore technical solutions to address this gap*.” (Interview 16)

Advanced clinical expertise was divided into three subcategories: expertise from allied health professionals (e.g., pharmacists, physiotherapists, social workers, and family therapists), gerontology, and palliative and hospice care. Palliative care, in particular, was identified as a critical area of expertise that significantly impacts work planning. There is a need to allocate resources for training and recruiting specialists in this field:

“*Palliative care certainly deserves recognition. It has gained attention as an essential area, and there′s a growing understanding that it requires dedicated resources. While there is some guidance on it now, it also demands adequate funding and specialized expertise*.” (Interview 17)

Comprehensive clinical knowledge was divided into four subcategories: care for diverse patient populations (e.g., children and adolescents, individuals with disabilities, substance abuse patients, and those with psychiatric conditions), in‐depth understanding of health and social care systems, skills for providing psycho‐social support, and the use of a broad spectrum of professional expertise. For instance, patients with long hospitalizations often require significant social, economic, and practical support to reintegrate into society. The importance of multidisciplinary expertise was also emphasized to meet these complex needs.

“*For instance, patients recovering from trauma (---), may need rehabilitation and social sector support, even with insurance coverage, to aid in their recovery and reintegration*.” (Interview 10)

#### 3.3.7. Autonomy in Evidence‐Based Practice

Autonomy in evidence‐based practice was divided into three generic categories: *Independent work ability*, *Professional role and self-development attitude*, and *Ability to assess and apply evidence-based practices*.

Independent work ability was divided into three subcategories: preference for autonomous work, both formal and informal qualifications, and the experience required for independent practice. The ability to work autonomously, without the constant need for a team, is crucial. Additionally, professional expertise and personal experience are both essential, as highlighted in one interview:

“*The role of a HaH care nurse is characterized by significant autonomy and responsibility. As a result, it demands a unique blend of experience-based knowledge, shaped by both professional expertise and personal experience*.” (Interview 2)

Professional role and self‐development attitude was divided into three subcategories: willingness to work independently in diverse and complex environments, and the ability to learn skills beyond one’s primary profession. There is a need for skills that extend beyond traditional in‐hospital care. Both physicians and nurses must demonstrate a readiness to acquire new skills outside their usual scope and adapt their existing expertise in a more holistic manner:

“*The opportunity to utilize a broad range of skills in this role, fostering a highly multiskilled workforce, undoubtedly contributes to job satisfaction*.” (Interview 2)

Ability to assess and apply evidence‐based practices was divided into four subcategories: care assessment, triage, cognitive and functional assessment, and evidence‐based decision‐making skills. In addition, participants emphasized the need for adequate financial compensation, as highlighted in one interview:

“*There are several specialized areas of expertise required for evidence-based practice and decision-making in the hospital-at-home setting. As a result, we hope these skills will be appropriately financially compensated*.” (Interview 17)

### 3.4. Synthesis of the Findings

Overall, the mixed‐methods findings differ yet complement one another: quantitative data identify concrete skill gaps, while qualitative insights emphasize wider, system‐level competencies to meet the evolving demands of home‐based care. Taken together, these complementary findings indicate that advancing the workforce will require a dual approach: addressing concrete skill deficits while simultaneously developing the broader competencies needed to meet the evolving demands of home‐based care.

## 4. Discussion

To the best of our knowledge, this is the first mixed method to explore future training and competency needs in HaH care from the perspectives of healthcare administrators and managers, encompassing not only nurses but all healthcare professionals. In future, HaH care requires a multidisciplinary team with varied expertise, and as the model continues to expand, new roles (e.g., “remote care coordinator,” “home safety assessment specialist”) and enhanced responsibilities will emerge to ensure high‐quality, patient‐centered care. This holistic approach shifts from rigid role‐specific responsibilities to a more comprehensive, competency‐based framework.

While the estimated competency levels were generally adequate, further training is necessary across all 23 predefined areas. In line with the previous literature, future training needs include end‐of‐life care, use of information systems, management of patients exhibiting disruptive behavior (including those in disability services), mental health and substance abuse expertise, and oral health care. However, the skills required to assess clients’ health status and service needs appear to be more advanced in home and long‐term care compared to HaH care [19].

According to our findings, future competency needs are related to Digital Health, Cultural and Linguistic Skills, Interprofessional Collaboration, Managerial Competence, Home Environment Safety, Clinical Expertise, and Autonomy in Evidence‐Based Practice. As home‐based healthcare expand, it is crucial to define, support, and evaluate the evolving competencies of healthcare professionals to meet the growing demands and ensure high‐quality, patient‐centered and professional care [23]. Previous studies also show that patient health and safety are directly influenced by the competency of healthcare providers [24].

Digital transformation in HaH care is in growing demand [25], but challenges persist due to a lack of incentives, digital strategies, and sustainable funding [13]. In line with previous research, digital health competencies remain relatively low [26], highlighting the urgent need to enhance healthcare professionals’ technological literacy [10] and competence in digital counseling [27].

In line with Rusli et al. [10], cultural and religious sensitivity is essential for healthcare professionals to effectively address the diverse needs of patients from various cultural, linguistic, and socioeconomic backgrounds. The importance of cultural and religious sensitivity is growing due to the increasing number of palliative and hospice care patients [28].

Interprofessional collaboration (e.g., interpersonal and interdisciplinary collaboration) is crucial for enhancing healthcare delivery [10, 29], especially in telemedicine [30]. In line with Tan et al. [31], registered nurses play a vital role in working with remote physicians to manage patients’ medical conditions through telephone or video consultations. Both nurses and physicians recognize the importance of building and maintaining trust, as physicians rely heavily on nurses for providing objective information to support clinical decision‐making.

Our findings provide new knowledge about managerial competencies in HaH care, while previous literature has primarily focused on nurses’ abilities to coordinate care [10, 29]. Consistent with Jordal et al. [31], managerial competencies are closely linked to organizational and economic responsibilities. While the managerial role encompasses numerous practical and technical tasks, such as workforce planning and staffing adjustments, the economic responsibilities are particularly significant. Furthermore, leading remote teams and overseeing staff well‐being—such as ensuring occupational safety during home visits—presents unique challenges compared to traditional in‐hospital settings [32].

The ability to work independently presents another unique challenge compared to traditional in‐hospital settings. Additionally, nurses and physicians must demonstrate greater autonomy and expand their skills beyond traditional roles [10, 29]. In line with Andersson et al. [1], a broader skill set is also required to effectively care for emerging patient groups, such as children, individuals with disabilities, and patients with psychiatric conditions.

## 5. Conclusion

The quantitative and qualitative findings show that workforce development in home‐based care requires both targeted skills training and broader competence strengthening. Quantitative results reveal specific, measurable gaps—particularly in RAI assessment, mental health care, substance abuse expertise, and managing disruptive behaviors—while qualitative insights point to wider, system‐level competencies essential for future practice, such as digital health proficiency, cultural and linguistic competence, interprofessional collaboration, managerial capabilities, home‐environment safety, clinical expertise, and autonomy in evidence‐based practice. Taken together, these complementary findings indicate that advancing the workforce will require a dual approach: addressing concrete skill deficits while simultaneously developing the broader competencies needed to meet the evolving demands of home‐based care.

### 5.1. Limitations

The study has several limitations that should be taken into account. First, the policies and organizational structures of HaH care, as well as the availability of related services, can vary significantly across different countries and municipalities, potentially affecting the generalizability of the results. In addition, the study was conducted from August to October 2023 and competency needs may undergo dynamic changes.

The perspective is somewhat limited, as it draws only on the views of healthcare administrators and managers, without input from other stakeholders such as nurses, family physicians, or patients and families. This may restrict a comprehensive understanding of training and competency needs. Nevertheless, the mixed‐methods design and the inclusion of administrators and managers—who provide valuable system‐level insights—remain key strengths.

Furthermore, the transcripts were not returned to participants for review or correction, and no field notes were taken during the interviews. However, as the transcripts were transcribed verbatim from the recordings, they can still be considered reliable sources of information.

## 6. Implications for Nursing Management

This study offers valuable insights for the nursing community, particularly for those in leadership and management roles. First, the findings underscore the growing complexity of HaH care and the increasing demand for advanced, specialized nursing competencies—highlighting the critical need for continuous professional development and role‐specific training. Home‐based care places more advanced demands on assessment and triage skills than in‐hospital care, highlighting the necessity for further targeted training.

Second, delivering truly integrated, multidisciplinary care requires a thorough understanding of the evolving training and competency needs across all healthcare disciplines involved in home‐based care. Nursing managers are uniquely positioned to champion and coordinate efforts that strengthen interprofessional collaboration. Interprofessional collaboration requires new methods that strengthen two‐way communication—such as structured communication protocols, shared digital documentation, and regular multidisciplinary meetings—to improve work distribution, enhance cooperation between service providers, and build trust between physicians and nurses in remote care setting.

Third, to adequately prepare future practitioners, nursing leadership must foster the development of flexible, forward‐looking educational programs. These programs should incorporate essential competencies—including digital health literacy, cultural and linguistic competence, interprofessional teamwork, clinical decision‐making, home‐environment safety, leadership, and autonomous evidence‐based practice. Collectively, these findings hold important implications for both curriculum development and policy‐making. Prior to this, the establishment of standardized frameworks and definitions is essential to ensure coherence and direction for curriculum development.

Finally, nursing managers play a pivotal role in establishing routine competency assessments and providing sustained mentorship and resources—ensuring healthcare professionals are well equipped to deliver safe, effective, and compassionate care in the home setting. This also requires the use of validated competence instruments to reliably assess practitioners’ skill levels and guide their ongoing professional development.

## Funding

This study was supported by the Government’s analysis, assessment, and research activities, Prime Minister’s Office, Finland (VN/31368/2022). Open access publishing facilitated by Oulun yliopisto, as part of the Wiley ‐ FinELib agreement.

## Disclosure

The funder has not influenced the design, conduct, analysis, or reporting of the study.

## Conflicts of Interest

The authors declare no conflicts of interest.

## Data Availability

The data that support the findings of this study are available upon request from the corresponding author. The data are not publicly available due to privacy or ethical restrictions.
